# A Novel Strategy for Mitigating Central Skin Depression and Dog-Ear Formation Following Suturing of Fusiform Incisions

**DOI:** 10.1007/s00266-025-04953-2

**Published:** 2025-05-23

**Authors:** Erlong Li, Qin Yang, Meirong Zou

**Affiliations:** 1 Department of Dermatology, West China Hospital, Sichuan University, Chengdu, China; 2 Laboratory of Dermatology, Clinical Institute of Inflammation and Immunology, Frontiers Science Center for Disease-related Molecular Network, West China Hospital, Sichuan University, Chengdu, China; 3 Department of Dermatology, The First People’s Hospital of Longquanyi District, No. 669, Longquan, donglang Road, Longquanyi District, Chengdu, 610100 Sichuan Province China

**Keywords:** Central skin depression, Dog-ear, Lozenge-shaped incision, Cutting method, Subcutaneous advancement flaps, Suspension sutures

## Abstract

*No Level Assigned* This journal requires that authors assign a level of evidence to each submission to which Evidence-Based Medicine rankings are applicable. This excludes Review Articles, Book Reviews, and manuscripts that concern Basic Science, Animal Studies, Cadaver Studies, and Experimental Studies. For a full description of these Evidence-Based Medicine ratings, please refer to the Table of Contents or the online Instructions to Authors www.springer.com/00266.

**Supplementary Information:**

The online version contains supplementary material available at 10.1007/s00266-025-04953-2.

Dear Editor,

We are writing this letter to disclose our finding: a surgical method to mitigate central depression and dog-ear formation.

## Clinical Challenge

The fusiform incision is widely employed for skin tumor excision. However, suturing such incisions may lead to two common complications: (1) central skin depression due to tissue loss at the midline and (2) dog-ear deformities caused by redundant tissue at the wound apices [1, 2]. These issues are particularly pronounced in convex anatomical regions, such as the forehead and zygoma. Achieving optimal esthetic outcomes while minimizing incision length remains a critical challenge in dermatologic surgery.

## Proposed Technique

**Fig. 1:**
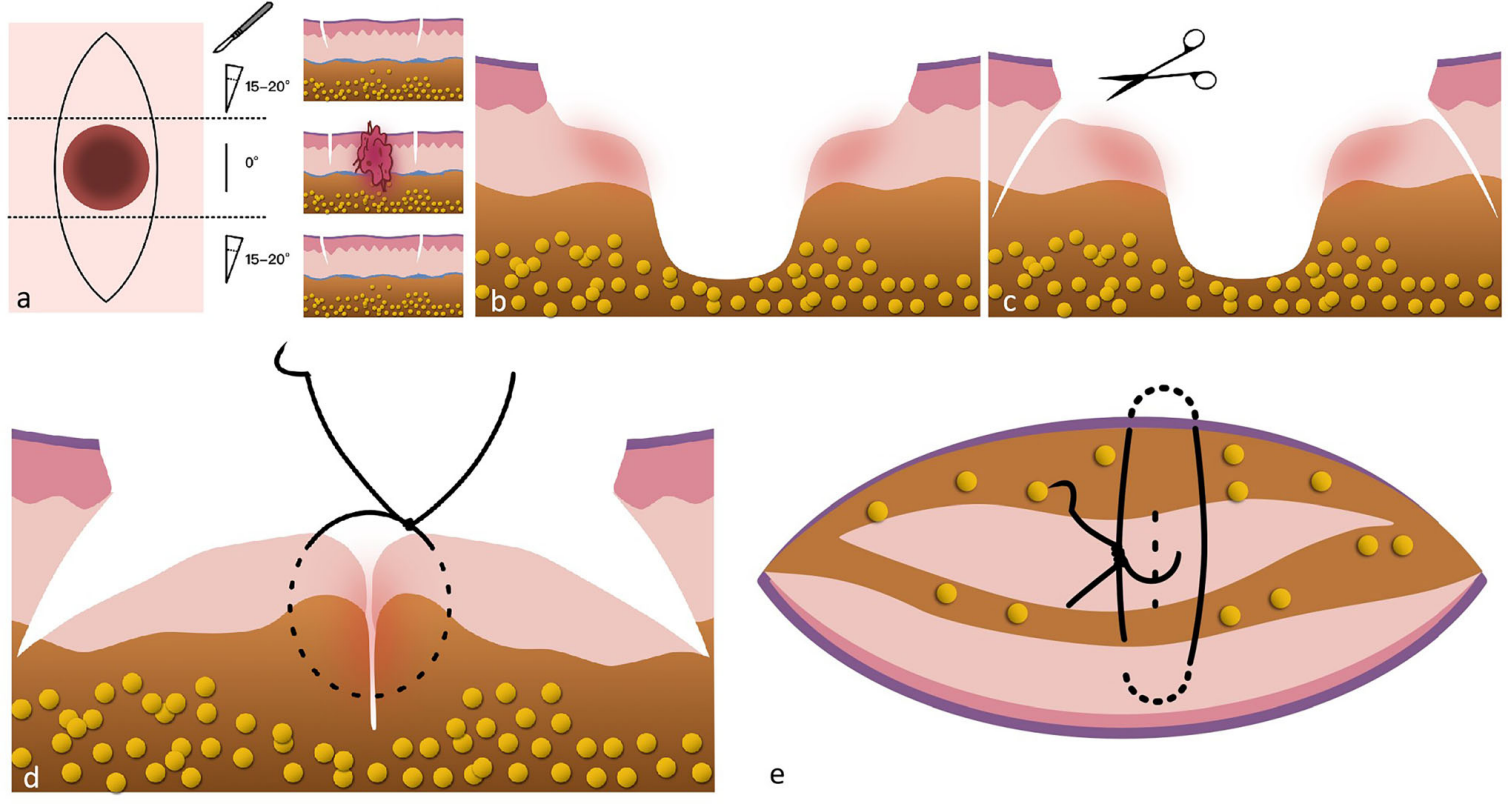
**a** Horizontal cross-sectional view: Angled (15–20°) excision at wound apices (dermal layer) versus perpendicular excision at the central defect (adipose layer). **b** Longitudinal section corresponding to **a**. **c** Subcutaneous tissue mobilization along wound edges. **d** Advancement of apical tissue to form flaps, secured with temporary sutures. **e** Suspension sutures connecting flaps to dermis in perpendicular planes

We introduce a modified surgical approach integrating subcutaneous advancement flaps and suspension sutures to address both central depression and dog-ear formation. The procedure is executed as follows:

1. Incision design: At the apices of the lozenge-shaped defect, the surgical blade is angled at 15–20° relative to the skin surface, creating an inverted trapezoidal excision extending to the dermal layer. At the central portion of the defect, the blade is oriented perpendicularly to the skin, penetrating through the adipose layer (Fig. 1a, b).
2. Subcutaneous tissue mobilization: Extensive undermining of subcutaneous tissue is performed along both wound edges to enhance tissue mobility (Fig. 1c).
3. Advancement flap creation: Excess subcutaneous tissue from the wound apices is advanced toward the central defect, forming bilateral advancement flaps. These flaps are temporarily secured with interrupted sutures (Fig. 1d).
4. Suspension suture placement: Without cutting the suture thread, suspension sutures are placed in two perpendicular planes within the dermal and subcutaneous layers. This technique anchors the advancement flaps to the dermis, eliminating dead space and reinforcing structural support (Fig. 1e).
5. Final closure: Standard tension-reducing sutures are utilized to complete the epidermal closure.

**Fig. 2:**
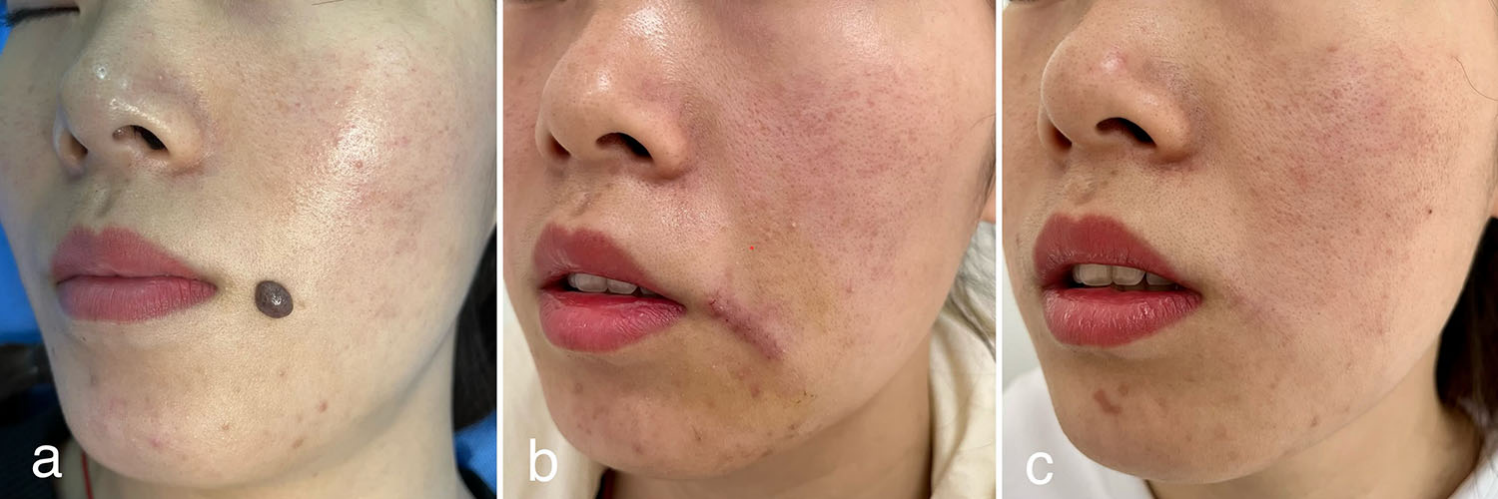
Oral labial melanocytic nevus (**a**). Postoperative appearance at 1 week (**b**). Postoperative outcome at 2 months (**c**)

**Fig. 3:**
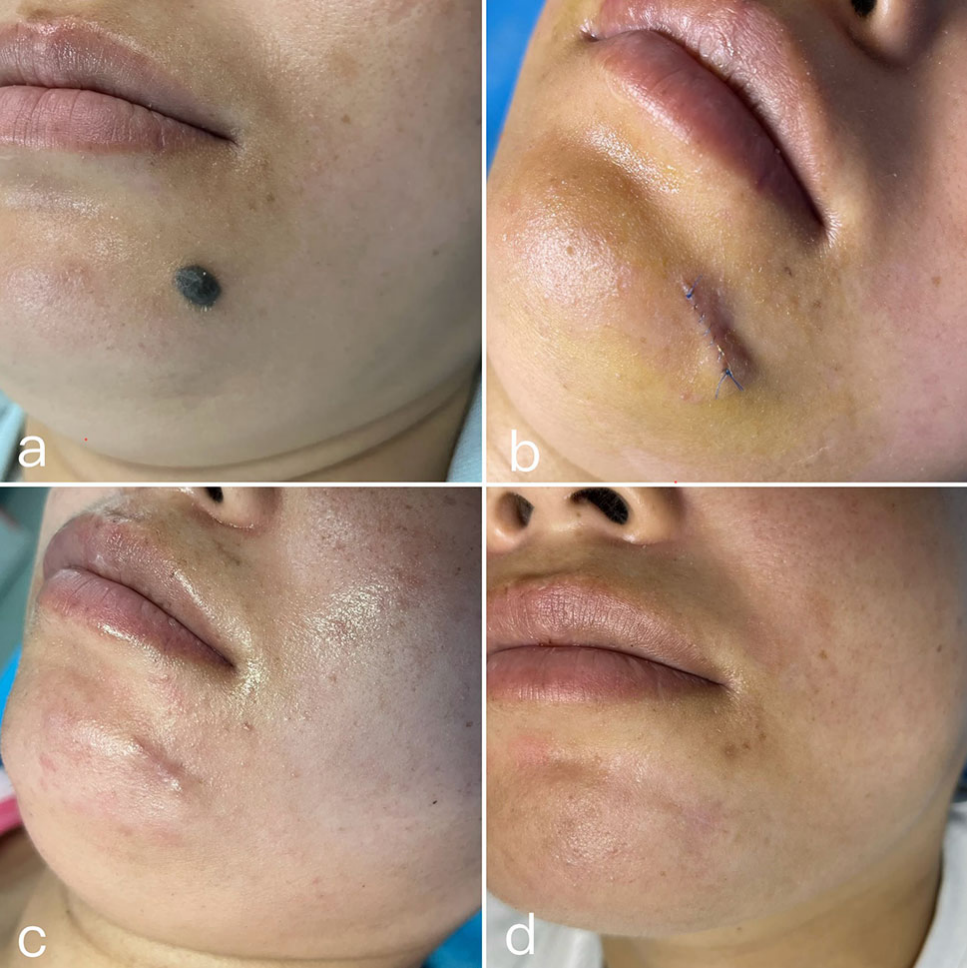
Mandibular melanocytic nevus (**a**). Postoperative status at 3 days (**b**). Postoperative appearance at 1 week (**c**). Postoperative result at 3 months (**d**)

Two cases with both immediate postoperative and long-term follow-up images were treated using this modified technique (Figs. 2, 3). Operational steps can be viewed in Video 1 and 2.

## Advantages

1. Dog-ear reduction: Angled excision at the apices minimizes redundant tissue, thereby reducing postoperative dog-ear formation.
2. Central depression correction: Advancement flaps compensate for midline tissue loss, preventing contour irregularities.
3. Dead space elimination: Suspension sutures secure the flaps and stabilize subcutaneous layers, reducing hematoma risk.

## Limitations

Not suitable for short incisions due to limited tissue availability. Contraindicated in thin-skin regions (e.g., periorbital area, anterior neck) where subcutaneous tissue is insufficient.

## Clinical Applicability

This technique is most effective in regions with adequate dermal and subcutaneous thickness, such as the zygoma, back, and buttocks. It is particularly advantageous for convex surfaces prone to dog-ear formation.

## Supplementary Information

Below is the link to the electronic supplementary material.

Supplementary Video 1: Demonstration of the technique on porcine skin. (MP4 446994 kb)

Supplementary Video 2: Clinical application in a patient with dermatofibroma on the lateral lower leg. (MP4 411105 kb)
